# Heart failure and levels of other comorbidities in patients with chronic obstructive pulmonary disease in a Swedish population: a register-based study

**DOI:** 10.1186/s13104-016-2008-4

**Published:** 2016-04-12

**Authors:** Elzbieta Kaszuba, Håkan Odeberg, Lennart Råstam, Anders Halling

**Affiliations:** Olofström Primary Health Care Centre, 293 32 Olofström, Sweden; Department of Clinical Sciences in Malmö, General Practice/Family Medicine, Lund University, 205 02 Malmö, Sweden; Research Unit of General Practice, Institute of Public Health, University of Southern Denmark, 5000 Odense, Denmark

**Keywords:** Heart failure, Chronic obstructive pulmonary disease, Diagnosis, Registries, Comorbidity, Primary care, Delivery of health care

## Abstract

**Background:**

Despite the fact that heart failure and chronic obstructive pulmonary disease (COPD) often exist together and have serious clinical and economic implications, they have mostly been studied separately. Our aim was to study prevalence of coexisting heart failure and COPD in a Swedish population. A further goal was to describe levels of other comorbidity and investigate where the patients received care: primary, secondary care or both.

**Methods:**

We conducted a register-based, cross-sectional study. The population included all people older than 19 years, living in Östergötland County in Sweden. The data were obtained from the Care Data Warehouse register from the year 2006. The diagnosis-based Adjusted Clinical Groups Case-Mix System 7.1 was used to describe the comorbidity level.

**Results:**

The prevalence of the diagnosis of heart failure in patients with COPD was 18.8 % while it was 1.6 % in patients without COPD. Age standardized prevalence was 9.9 and 1.5 %, respectively. Standardized relative risk for the diagnosis of heart failure in patients with COPD was 6.6. The levels of other comorbidity were significantly higher in patients with coexisting heart failure and COPD compared to patients with either heart failure or COPD alone. Primary care was the only care provider for 36.2 % of patients with the diagnosis of heart failure and 20.7 % of patients with coexisting diagnoses of heart failure and COPD. Primary care participated furthermore in shared care of 21.5 % of patients with the diagnosis of heart failure and 21.7 % of patients with coexisting diagnoses of heart failure and COPD. The share of care between primary and secondary care varied depending on levels of comorbidity both in patients with coexisting heart failure and COPD and patients with heart failure alone.

**Conclusion:**

Patients with coexisting diagnoses of heart failure and COPD are common in the Swedish population. Patients with coexisting heart failure and COPD have higher levels of other comorbidity than patients with heart failure or COPD alone. Primary care in Sweden participates to a great extent in care of patients with diagnoses of heart failure alone and coexisting heart failure and COPD.

## Background

Worldwide, chronic obstructive pulmonary disease (COPD) is one of the most common chronic diseases with overall prevalence of 7.6 % [1] and a heavy economic burden [2]. Patients with COPD are a group where chronic cardiovascular diseases including heart failure occur more frequently than in the general population [3]. The risk of developing heart failure in patients with COPD is 4.5 times higher than in age-matched controls [4]. Coexisting heart failure and COPD can be overlooked due to similarities in symptoms and signs, which is an important clinical implication. The main clinical manifestation of COPD and heart failure is dyspnea, which in turn is one of the most common causes of consultations in both primary and secondary care, especially among elderly patients [5].

The prevalence of undiagnosed heart failure in patients with COPD older than 65 years in primary care is approximately 20 % [6]. A review of previous studies showed that the prevalence of heart failure in patients with COPD varied between 10 and 46 % [7].

The prevalence of COPD in patients with heart failure is also about 20 % [8, 9]. The prevalence and burden of heart failure and COPD correlate with an aging population. The proportion of elderly in Sweden is the highest in the world with 17.4 % of persons aged ≥65 years and is expected to increase until the year 2020 [10]. Management of heart failure and COPD is going to be a challenge in both primary and secondary care. Despite the fact that heart failure and COPD often occur together and have serious clinical and economic implications, both diseases have so far been mostly studied separately, especially on the population level. There is no Swedish data about prevalence of coexisting heart failure and COPD.

The aim of this study was to examine the prevalence of the diagnosis of heart failure in patients with the diagnosis of COPD in the Swedish population. A further aim was to describe the levels of other comorbidities and investigate where patients with coexisting heart failure and COPD receive care: primary, secondary care or both.

## Methods

This was a register-based, cross-sectional study. All the study population included people older than 19 years, living in Östergötland County in Sweden. The data used for the study was not openly available and was obtained after permission from the Östergötland County council. We used data from the Care Data Warehouse register [11]. The register collects data concerning consultation and diagnosis transferred every month from all public and private health care units in both primary and secondary care. Diagnoses were recorded according to the Swedish Version of International Statistical Classification of Diseases and Related Health Problems version 10 (ICD 10). We used data from the year 2006. We identified an individual as having heart failure or COPD if the diagnosis code I50 or J44 was recorded on at least one consultation in primary or secondary care including hospitalization.

The code I50 covers: I50.0-chronic heart failure including congestive heart failure, right heart failure secondary to left heart failure, I50.1-left ventricular failure with or without lung oedema and asthma cardiale, I50.9 heart failure, unspecified.

The code J44 comprised the following: J44 chronic obstructive lung disease, J44.0 chronic obstructive lung disease with acute infection in lower airways, J44.1 chronic obstructive lung disease with acute exacerbation, unspecified, J44.8 other specified chronic obstructive lung disease including chronic bronchitis with emphysema.

The diagnosis-based Adjusted Clinical Groups (ACG) Case-Mix System 7.1 was used to describe comorbidity [12, 13]. Comorbidity on an individual level was measured when the diagnoses I50 and J44 were excluded and it is referred to as the level of other comorbidity.

Each individual was assigned one of six comorbidity levels called resource utilization bands (RUB) graded from 0 to 5.

When identifying the place where patients received care we used information where the diagnosis of heart failure and COPD was made: primary, secondary care or both.

### Statistics

Data were analyzed in the STATA version 10 (Stata Corporation, Texas, USA). Descriptive data were presented in tables. Differences in proportions between the groups were tested using the Chi square test. A p < 0.05 was considered significant. Results for prevalence of heart failure and COPD are given for the whole study population. Thereafter direct standardizing for age was made. Individuals aged 60 and above were chosen arbitrarily as a standard population.

### Ethics

The study has been approved by the Research Ethics Committee at Linköping University No 147/05 and 29/06.

## Results

The study population consisted of 313,977 individuals. The diagnosis of heart failure was registered in 1.8 % and the diagnosis of COPD was registered in 1.2 % of the study population. The mean age in patients with the diagnosis of heart failure was 78.4 years (CI 78.0–78.7). The mean age of patients with the diagnosis of COPD was 70.5 years (CI 70.2–70.9). The prevalence of both diagnoses increased with age (Table 1). The prevalence of the diagnosis of heart failure in patients with the diagnosis of COPD was 18.8 and 1.6 % in patients without COPD. After standardizing for age the prevalence was 9.9 and 1.5 %, respectively. Standardized relative risk for the diagnosis of heart failure in patients with COPD was 6.6.

**Table 1:** Prevalence of diagnoses COPD, heart failure and coexisting COPD and heart failure in the study population

Variable	20–39 years	40–59 years	60–79 years	>80 years	Total
Women	47,419	53,539	41,359	16,386	158,703
Men	52,020	55,164	38,443	9647	155,274
COPD in total population	31 (0.03 %)	536 (0.4 %)	2280 (2.8 %)	889 (3.4 %)	3736 (1.2 %)
Heart failure in total population	34 (0.03 %)	328 (0.3 %)	2132 (2.6 %)	3045 (11.6 %)	5539 (1.89 %)
Heart failure in patients with COPD	0 (0 %)	26 (4.6 %)	367 (16.1 %)	307 (34.5 %)	700 (18.8 %)
Heart failure in patients without COPD	34 (0.03 %)	302 (0.3 %)	1765 (2.3 %)	2738 (10.9 %)	4839 (1.6 %)

**Fig. 1:**
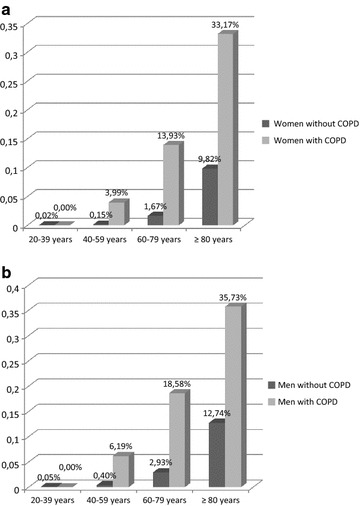
Prevalence of the diagnosis of heart failure in female (**a**) and male (**b**) patients with and without coexisting COPD in different age groups

The prevalence of the diagnosis of heart failure increased with age in women and men in both groups and reached 35.7 % in men with COPD ≥80 years (Fig. 1). The prevalence of the diagnosis of heart failure was significantly higher in both women and men with COPD comparing to women and men without COPD in all age groups apart from the age group 20–39 years.

The most common comorbid condition in all three groups of patients: heart failure alone, COPD alone and coexisting heart failure and COPD was essential (primary) hypertension coded with either I10 or I10.9. The latter code is used only in secondary care. The comorbid diagnoses are summarized in Table 2.

**Table 2:** Summarizing of the most common diagnostic codes

Patients with COPD n = 3736	Code	Number (%)	Patients with heart failure n = 4839	Code	Number (%)	Patients with COPD and heart failure n = 700	Code	Number (%)
Essential (primary) hypertension	I10, I10.9	1196 (32)	Essential (primary) hypertension	I10, I10.9	2303 (47.6)	Essential (primary) hypertension	10, I10.9	284 (40.6)
Observation for suspected disease or condition	Z039	628 (16.8)	Observation for suspected disease or condition	Z03.9	1472 (30.4)	Observation for suspected disease or condition	Z03.9	251 (35.9)
Heart failure, unspecified	I50.9	526 (14.1)	Atrial fibrillation and atrial flutter, unspecified	I48.9	1380 (28.5)	Atrial fibrillation and atrial flutter, unspecified	I48.9	199 (28.4)
Unspecified acute lower respiratory infection	J22	383 (10.3)	Type 2 diabetes mellitus, without complications	E11.9	958 (19.8)	Old myocardial infarction	I25.2	166 (23.7)
Type 2 diabetes mellitus, without complications	E11.9	350 (9.4)	Atrial fibrillation and atrial flutter, unspecified	I48.9	931 (19.2)	Type 2 diabetes mellitus, without complications	E11.9	149 (21.3)
Atrial fibrillation and atrial flutter, unspecified	I48.9	312 (8.4)	Chronic ischaemic heart disease, unspecified	I25.9	910 (18.8)	Chronic ischaemic heart disease, unspecified	I25.9	136 (19.4)

The levels of other comorbidity were significantly higher in patients with coexisting heart failure and COPD comparing to patients with heart failure or COPD alone (Table 3).

**Table 3:** Distribution of levels of other comorbidity measured by RUB (resource utilization band) in the study population

	Total	Patients without COPD		Patients with COPD		p value*
		No heart failure	Heart failure	No heart failure	Heart failure	
RUB 0	102,071 (32.51 %)	101,835 (33.34 %)	81 (1.65 %)	150 (4.49 %)	5 (0.71 %)	0.000
RUB 1	44,126 (14.05 %)	43,855 (14.36 %)	86 (1.78 %)	181 (5.96 %)	4 (0.57 %)	0.001
RUB 2	65,322 (20.8 %)	64,533 (21.13 %)	282 (5.83 %)	481 (15.84 %)	26 (3.71 %)	0.000
RUB 3	89,042 (28.36 %)	84,602 (27.7 %)	2514 (51.95 %)	1603 (52.80 %)	323 (46.14 %)	0.000
RUB 4	10,383 (3.31 %)	10,383 (3.31 %)	1234 (25.50 %)	434 (14.30 %)	203 (29 %)	0.000
RUB 5	3033 (0.97 %)	2065 (0.68 %)	642 (13.27 %)	187 (6.16 %)	139 (19.89 %)	0.000
Total	313,977 (100 %)	305,402 (100 %)	4839 (100 %)	3036 (100 %)	700 (100 %)	0.000

* p value describes differences between groups with and without COPD

The proportion of individuals with higher levels of other comorbidity (RUB 3–5) was 95 % in the group with coexisting heart failure and COPD, while in the group with heart failure alone it was 90.7 % and in the group with COPD alone it was 73.3 %.

Primary care was the only care provider to 36.2 % of patients with the diagnosis of heart failure and 20.7 % of patients with coexisting heart failure and COPD. Furthermore, primary care participated in shared care of 21.5 % of patients with the diagnosis of heart failure alone and 21.7 % of patients with coexisting diagnoses of heart failure and COPD.

**Fig. 2:**
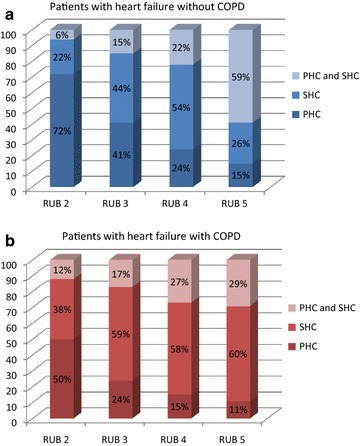
Share of care in patients with heart failure without (**a**) and with (**b**) chronic obstructive pulmonary disease. *PHC* primary health care, *SHC* secondary health care, *RUB* resource utilization band

The share of care given by primary and secondary care varied depending on levels of other comorbidity both in patients with heart failure without COPD and patients with coexisting heart failure and COPD (Fig. 2). The higher the level of other comorbidity the larger the participation of secondary health care was. Among patients with the highest level of other comorbidity (RUB 5) in the group with coexisting heart failure and COPD 11 % received care only in primary care, 29 % received shared care and 60 % received care only in secondary care.

## Discussion

We found that the prevalence of the diagnosis of heart failure in patients with the diagnosis of COPD was significantly higher than in patients without COPD. The levels of other comorbidity were significantly higher in patients with coexisting heart failure and COPD comparing with patients with heart failure or COPD alone. Primary care alone delivered care to 20.7 % of patients with coexisting heart failure and COPD and to a further 21.7 % who simultaneously received care from primary and secondary care.

### Completeness and accuracy of data

The Care Data Warehouse Register comprised data from the whole population in the Östergötland County, which is the strength of our study. All consultations were recorded in electronic charts and the diagnosis was required at each consultation. We analyzed data from both primary and secondary care. A previous study showed usefulness of data from the Care Data Warehouse register in Östergötland in estimating the prevalence of chronic diseases such as diabetes, hypertension, ischemic heart disease, asthma and COPD and showed that the longer period of analysis corresponded to better capturing of cases [14]. The limitation of our study is a short period we analyzed. Our choice was anchored in Swedish clinical practice. In Swedish primary care patients with chronic diseases are actively checked at least once a year. Patients with COPD are summoned for a nurse-led follow-up and the presence of a specially educated asthma/COPD nurse is a requirement for each primary health care centre.

Nurse-led follow up of patients with heart failure is common in Swedish secondary care [15]. Östergötland was the leading county in Sweden in structured heart failure management in primary care [16].

A previous study showed that the diagnosis of COPD from a register in Sweden has an acceptable validity for being used in epidemiological research [17]. COPD in Sweden is usually diagnosed in primary care according to the Global Initiative for Chronic Obstructive Lung Disease (GOLD) criteria [18]. Spirometry as a gold standard in diagnosing COPD is widely available in primary care [19, 20]. Echocardiography as a gold standard in diagnosing heart failure is not as much accessible to general practitioners as spirometry and requires referral to specialized care. A previous Swedish study showed that echocardiography was performed only in about 30 % of patients with suspected heart failure [21]. Östergötland County was a leader in Sweden in use of natriuretic peptides in patients with suspected heart failure in primary care [22]. The limitation of our study was that, due to ethical reasons, we could not validate recorded diagnoses against the original medical records. At the same time we have no premises to suspect that the diagnoses registered in the Care Data Warehouse were non-accurate.

### Interpretation of the results

We found that the diagnosis of heart failure occurred in 1.8 % of the study population.

This is exactly the same as the crude prevalence reported in a recently published Swedish register study [23]. According to this study the estimated prevalence of heart failure in Sweden was 2.2 % in year 2010. Estimation made about 15 years ago was 2.5 % [24].

The prevalence of the diagnosis of COPD was 1.2 %. Our result is considerably lower than prevalence of COPD in the general population in Sweden estimated at about 6 % [25]. The discrepancy was expected because epidemiological studies regarding COPD used to be performed in individuals aged at least 40 years due to the onset of the illness, while we included individuals aged 20 and older in order to study the whole adult population. After excluding younger patients aged ≤40 years the prevalence of COPD was 1.7 %, which did not change our results considerably.

We searched only for the diagnostic code J44, while in studies from other countries chronic bronchitis and emphysema J40–J43 were also included [26]. By that choice we wanted to decrease a risk of the non-accurate COPD diagnosis. In view of present guidelines for the diagnosis of COPD in Sweden the diagnostic code J44 is expected to be used for patients with COPD after doing the spirometry. This code is expected even in patients with emphysema caused by COPD. We think that prevalence of COPD in our study was underestimated due to poor registering of the diagnosis code in medical records.

Prevalence of the diagnosis of heart failure increased with age in patients without and with COPD as reported in previous studies [27, 28]. In our study the prevalence of the diagnosis of heart failure in individuals ≥80 years in the general population was approximately 11 % and was comparable with previously reported data from epidemiological studies, but differed from the recent Swedish register study where it was about 19 % [23]. A difference between genders was reported in a Swedish epidemiological study. A report from 2001 [29] showed that heart failure was more prevalent in men up to 80 years of age, thereafter, heart failure was more prevalent in women. Our data from 2006 showed no significant difference between women and men even aged ≥80 years. The newer study [23] with data collection up to 2010 showed, in contrast to the first study, that prevalence was higher in men in groups 80–89 and 90–99.

Women dominated slightly in the group ≥100 years. The populations in all three studies were large enough to venture to assert that the age limit for healthy survivals has moved during one decade to centenarians, probably thanks to improved strategies in heart failure management in Sweden.

The diagnosis of heart failure occurred in 18.8 % of patients with COPD while in patients without COPD it occurred in 1.6 %. Due to differences in age between groups with and without COPD standardizing for age was made and standardized prevalence was 9.9 % in patients with COPD and 1.5 % in patients without COPD. Previously reported prevalence of heart failure in patients with COPD varied between 10 and 46 % [7]. These data were obtained in different settings and different procedures were used to make the diagnosis of heart failure, ranging from clinical symptoms, standardized chronic heart failure score, the use of natriuretic peptides for assessment of left and right ventricle function by ventriculography or echocardiography. The highest prevalence was reported in emergency setting among patients with symptomatic dyspnea [30]. The diagnosis of heart failure in this study was established by assessment of left and right ventricle function by radionuclide ventriculography. The prevalence of the diagnosis of heart failure found in our study (18.8 %) is in agreement with the prevalence of heart failure in patients with COPD (20.5 %) found in the Dutch study conducted in primary health care setting [6]. The diagnosis of heart failure in that study was made by using echocardiography. In our register-based study we could not trace how the diagnosis of heart failure was made.

Differences in frequency of the diagnosis of heart failure between individuals with and without COPD were significant in all age groups except the age 20–39. Calculations in this age group were affected by 0 % prevalence of heart failure diagnosis in individuals with COPD.

Comorbidity in patients with heart failure and COPD is well-known and widely reported [31–37]. The most common comorbid condition in all three groups of patients in our study: COPD alone, heart failure alone and coexisting heart failure and COPD was essential (primary) hypertension coded with either I10 or I10.9. The latter code is used only in secondary care.

The next common diagnosis code in all three groups was Z03.9 Observation for suspected disease or condition, unspecified. This code does not allow identifying of disorder. If the register data should be available as a source of research e.g. prevalence studies, more specific diagnosis codes are desirable. Comorbidity should be considered as an important factor when analyzing needs for health care resources. ACG Case Mix is able to describe comorbidity in a quantitative way and is used in the Swedish health care system for calculation of payment rates. When analyzing comorbidity levels we excluded the main diagnoses of heart failure and COPD. This was done in order to study the importance of other comorbidity that otherwise would be hidden by the heavy burden connected with the diagnosis of heart failure or COPD. The levels of other comorbidity calculated in this way were significantly higher in patients with coexisting heart failure and COPD. That implies a greater need for health care and more extensive resource utilization than in patients with only heart failure or COPD.

The delivery of care to patients with coexisting heart failure and COPD has not been studied previously. When presenting our results in Fig. 2 we omitted groups with the lowest levels of other comorbidity (RUB 0 and 1) due to a small number of patients in those groups. As expected, the higher the levels of other comorbidity the greater participation of secondary care. We did not trace what kind of care patients got in secondary health care units. It might be hospitalizations or consultations in specialized outpatient clinics. Taking into consideration the heavy burden of both heart failure and COPD the participation of primary care was large in our opinion. Half of the patients with coexisting heart failure and COPD and lower levels of other comorbidity (RUB 2) received care only in primary care. In the group with the highest level of other comorbidity (RUB 5) and coexisting heart failure and COPD primary care was involved in almost 40 % of patients, either alone or together with secondary care. In the case of patients with heart failure without COPD the percentage was even higher (74 %). Shared care can be explained by organization of the Swedish health care system and established co-operation between primary and secondary care units concerning chronic diseases.

It is noteworthy that a considerable part of patients with the highest level of other comorbidity (RUB 5) received care only in primary care. A possible explanation might be that the patients were optimally treated and requirement of secondary care was unnecessary during one year period we analyzed. It might also be a matter of the terminal phase of illness and palliative care where primary care plays a central role.

## Conclusion

Our study showed that the prevalence of the diagnosis of heart failure in patients with the diagnosis of COPD is common in the Swedish population. Patients with coexisting heart failure and COPD have higher levels of other comorbidity than patients with heart failure or COPD alone. Primary care in Sweden participates to a great extent in care of patients with heart failure and coexisting COPD as well as without COPD.

## Abbreviations

COPD: chronic obstructive pulmonary disease
ACG: adjusted clinical groups,
RUB: resource utilization band
PHC: primary health care
SHC: secondary health care
